# Sperm Aneuploidy in Faroese Men with Lifetime Exposure to Dichlorodiphenyldichloroethylene (*p*,*p´*-DDE) and Polychlorinated Biphenyl (PCB) Pollutants

**DOI:** 10.1289/ehp.1509779

**Published:** 2015-11-04

**Authors:** Melissa J. Perry, Heather A. Young, Philippe Grandjean, Jónrit Halling, Maria Skaalum Petersen, Sheena E. Martenies, Parisa Karimi, Pál Weihe

**Affiliations:** 1 Department of Environmental and Occupational Health, and; 2 Department of Epidemiology and Biostatistics, Milken Institute School of Public Health, George Washington University, Washington, DC, USA; 3 Department of Environmental Medicine, University of Southern Denmark, Odense, Denmark; 4 Department of Environmental Health, Harvard T.H. Chan School of Public Health, Boston, Massachusetts, USA; 5 Department of Occupational Medicine and Public Health, Faroese Hospital System, Tórshavn, Faroe Islands

## Abstract

**Background:**

Although it is known that sperm aneuploidy contributes to early pregnancy losses and congenital abnormalities, the causes are unknown and environmental contaminants are suspected.

**Objectives:**

Our goal was to evaluate associations between lifetime exposure to organochlorines, specifically dichlorodiphenyldicholorethylene (p,p´-DDE) and polychlorinated biphenyls (PCBs), and sperm aneuploidy in men from the general population of the Faroe Islands, a population with a known history of organochlorine exposures.

**Methods:**

Serum and semen samples from men (n = 90) 22–44 years old who participated in Faroe Islands health studies were analyzed for p,p´-DDE and PCBs 118, 138, 153, and 180 and adjusted for total lipids. Cord blood and age-14 serum were available for a subgroup (n = 40) and were also analyzed for p,p´-DDE and PCBs. Sperm fluorescence in situ hybridization (FISH) for chromosomes X, Y, and 18 was used to determine rates of XX18, XY18, YY18, and total disomy. Multivariable adjusted Poisson models were used to estimate the relationship between organochlorine exposure and sperm disomy outcomes.

**Results:**

Adult p,p´-DDE and total PCB serum concentrations were both associated with significantly increased rates of XX18, XY18, and total disomy. Age-14 p,p´-DDE and PCB concentrations were both associated with significantly increased rates of XX, XY, and total disomy in adulthood. Associations between cord blood concentrations of p,p´-DDE and PCBs and sperm disomy in adulthood were not consistently significant.

**Conclusions:**

Organochlorine exposures measured at age 14 and in adulthood were associated with sperm disomy in this sample of high-exposure men, suggesting that the impacts of persistent pollutants on testicular maturation and function require further investigation.

**Citation:**

Perry MJ, Young HA, Grandjean P, Halling J, Petersen MS, Martenies SE, Karimi P, Weihe P. 2016. Sperm aneuploidy in Faroese men with lifetime exposure to dichlorodiphenyldichloroethylene (p,p´-DDE) and polychlorinated biphenyl (PCB) pollutants. Environ Health Perspect 124:951–956; http://dx.doi.org/10.1289/ehp.1509779

## Introduction

Environmental endocrine-disrupting chemicals have been associated with impaired male reproductive function (Bergman et al. 2013; Woodruff et al. 2008) and may affect sperm development (Schiffer et al. 2014). Aneuploidy, that is, an abnormal chromosome complement in the fetus, is thought to contribute to ≤ 50% of early pregnancy losses (Hassold et al. 2007). The most common aneuploidies in humans at birth involve an abnormal number of X or Y chromosomes (Hassold and Hunt 2001), and ≥ 50% of XXY trisomies originate from the father (Hassold et al. 2007). European data from consecutive birth studies show that the incidence of Klinefelter syndrome (XXY) appears to be increasing, but no increases have been observed in the incidence of XXX or XYY trisomies (Morris et al. 2008). Because XXY trisomies frequently arise from nondisjunction of the XY (paternal) bivalent during meiosis I, and increases in XXX trisomy (predominately maternally derived) have not been observed, underlying environmental causes affecting nondisjunction during spermatogenesis are suspected.

Because of their lipophilic nature and persistence, organochlorine contaminants biomagnify in the food chain [Agency for Toxic Substances and Disease Registry (ATSDR) 2008] and occur at measurable levels in a large proportion of the general population [Centers for Disease Control and Prevention (CDC) 2009]. These compounds transcend the blood–testis barrier and can alter the endocrine homeostasis essential for testicular function (Schiffer et al. 2014; Woodruff et al. 2008). In epidemiologic studies, organochlorine exposures have been associated with decreased semen quality (reviewed by Meeker and Hauser 2010) and miscarriage (Eskenazi et al. 2009; Toft 2014; Venners et al. 2005).

Environmental chemicals including nonpersistent pesticides (reviewed by Perry 2008) and benzene (Xing et al. 2010) have been associated with sperm aneuploidy in epidemiologic studies. Because it is the most frequent type of sperm aneuploidy (Hassold and Hunt 2001), most environmental exposure studies have focused on sex-chromosome disomy, which involves an extra X or Y chromosome. To our knowledge, only one previously published study has investigated the relationship between organochlorine compounds and human sperm sex-chromosome disomy (McAuliffe et al. 2012a). Among men recruited from a hospital-based fertility clinic, significant positive associations were found between dichlorodiphenyldicholorethylene (*p,p*´-DDE) and polychlorinated biphenyls (PCBs) and sex-chromosome disomy. There was a significant inverse association between PCBs and XX disomy. The aim of the present study was to investigate whether environmental exposure to *p,p*´-DDE and PCBs (118, 138, 153, and 180) was associated with sperm sex-chromosome disomy in a group of adult men who had elevated lifetime exposures to these pollutants. Because of environmental contamination, particularly from a seafood-rich diet including pilot whale, the population of the Faroe Islands is exposed to higher-than-average levels of persistent organic pollutants compared with other populations (Longnecker et al. 2003; Weihe and Joensen 2012). This population provides a unique context for cross-sectional and prospective investigations of pollutant exposures and male reproductive functioning among men recruited from communities rather than from clinical settings.

## Methods

### Study Participants

***Study population and recruitment.*** The study population consisted of adult men from the general Faroese population. Study population recruitment practices and inclusion criteria have been described previously (Halling et al. 2013; Petersen et al. 2015). Briefly, the parent study population consisted of 747 Faroese men recruited from three study groups: a birth cohort, a group of fertile men, and a group of men selected randomly from the population register. The birth cohort was established in the Faroes in 1986–1987. Biological samples and information on physical health and environmental exposures were obtained at birth and during follow-up examinations for cohort members at ages 14 and 22. Two hundred forty men participating in the 22-year follow-up from the original birth cohort agreed to participate in a study of semen quality and were examined in 2009–2010 (Halling et al. 2013).

The second group consisted of 266 proven fathers to children in a new Faroese birth cohort established in 2007–2009. Men were excluded if the pregnancy was achieved using fertility treatment (Petersen et al. 2015).

The third study group was recruited from men consecutively listed in the population register as being born between January 1981 and December 1984. Two hundred forty-one men from this group agreed to participate in a cross-sectional semen quality study and were examined between 2007 and 2009 (Halling et al. 2013).

The parent studies were approved by the local Science Ethics Committee for the Faroe Islands and the Institutional Review Board of the Harvard T.H. Chan School of Public Health. All subjects signed an informed consent form before participation. The study reported herein focused on a subsample of men identified from semen samples available from the parent studies. Three men from the birth cohort, 3 men from the fathers cohort, and 21 men from the population register did not provide semen samples. To be included in the birth cohort, men also needed to have had exposure data from cord blood and age-14 samples. Ninety samples were randomly selected from the available samples without respect to demographic or semen parameter characteristics and without regard to the parent study. Semen samples were analyzed using sperm fluorescence *in situ* hybridization (FISH) analysis (birth cohort *n* = 40; fathers cohort *n* = 12; population register *n* = 38). For men recruited from the birth cohort, data were also available on *in utero* exposures via cord blood samples for *n* = 40 subjects and exposures at age 14 for *n* = 33 subjects.

***Semen samples and analysis.*** Semen analysis methods have been described previously (Halling et al. 2013). Briefly, at the time of study recruitment, the men produced a semen sample via masturbation in a private room at the laboratory. Abstinence time was recorded at the time of sample collection. Three technicians trained in quality control measures at the National University Hospital (Rigshospitalet) in Denmark conducted all of the semen analysis for evaluation by World Health Organization (WHO) guidelines (WHO 1999). Semen values were dichotomized on the basis of reference values for three main semen quality parameters: specifically, these parameters are sperm count (< 15 million sperm per milliliter of ejaculate), motility (< 40% total motile sperm), and morphology (< 4% normal forms) (WHO 2010).

### Sperm Disomy Analysis

To detect sex-chromosome disomy (aneuploidy involving an extra X or Y chromosome), a single investigator blinded to exposure status performed FISH analysis. The procedures for this analysis have been described in detail previously (McAuliffe et al. 2012a). Briefly, the FISH procedure was performed for three chromosomes of interest, X, Y, and 18 (autosomal control), with a series of nonoverlapping field images taken for each prepared FISH slide using a fluorescence laser scanning wide-field microscope. Sex-chromosome disomy was the primary outcome of interest because of its reproductive health impacts: *a*) it is the most frequent form of sperm aneuploidy and occurs twice as frequently as autosomal disomy; *b*) sperm that are disomic for X or Y are capable of fertilization; and *c*) sex-chromosome disomy results in viable offspring. The images were scored with custom MATLAB software designed to utilize scoring criteria for size and shape as reported by Baumgartner et al. (1999). A colocalization analysis allowed the software to identify sperm nuclei and the number of signals contained therein. The method has been shown to produce results quantitatively and qualitatively comparable to manual scoring (Perry et al. 2007, 2011).

### Exposure Analysis

A two-stage solid-phase extraction method followed by gas chromatography analysis with electron capture detection was used to quantify the four most prevalent PCB congeners, CB-118, CB-138, CB-153, and CB-180 (Grandjean et al. 1995; Petersen et al. 2006), along with *p,p´-*DDE. The concurrent and age-14 results were adjusted for total serum lipid content and reported as micrograms per gram lipid, and the cord blood concentrations were expressed in terms of volume of whole blood (Grandjean et al. 2012). As in a previous study (McAuliffe et al. 2012a), PCB exposure was represented by the sum of the serum concentrations of the four most prevalent PCB congeners (118, 138, 153, and 180), Σ_4_PCBs. The median limit of detection (LOD) was 0.03 μg/L, which, at a mean lipid concentration of 7.45 g/L, corresponds to 0.004 μg/g lipid. Nondetectable levels of PCB congeners and *p,p´*-DDE were assumed to equal 0.002 μg/g lipid; this level corresponds to one-half the LOD.

### Statistical Analysis

Descriptive statistics for demographic and semen parameters were summarized using frequency distributions or means and standard deviations, as appropriate. Descriptive statistics for exposures and disomy data were summarized as means and standard deviations, geometric means, medians, and relevant percentiles. Poisson regression (SAS GENMOD procedure) was used to model the association between each of the disomy measures and organochlorine exposures (*p,p´*-DDE and Σ_4_PCBs) in unadjusted and adjusted analyses.

The number of sperm scored and the number of disomic nuclei identified were summed for each subject and used as the unit of analysis. In Poisson regression, the offset variable allows for control of time/space variation in the denominator. In this study, the source of variation was the number of nuclei scored per subject. The Poisson model was fitted using each disomy measure (XX18, YY18, XY18, or total sex-chromosome disomy) as the outcome variable, the natural logarithm of the number of sperm counted as the offset variable, and the organochlorine exposure of interest as the independent variable, with age, abstinence time, smoking status, log(sperm concentration), motility, and morphology as potential confounders in the adjusted analyses. Covariates were identified based on *a priori* considerations (Blackwell and Zaneveld 1992; Hassan and Killick 2003; Vine 1996) and were retained in the final models based on potential associations with the disomy outcomes and the organochlorine exposures. Sperm concentration, motility, and morphology were adjusted for because these variables have been associated with disomy in prior studies (e.g., Martin et al. 2003; McAuliffe et al. 2012b; Vegetti et al. 2000). Each was found to be a significant independent predictor of disomy outcomes, and all were retained in the final models. Because semen concentrations were skewed and residuals were not normally distributed, these values were natural log–transformed before being included in the models. Age, log(sperm concentration), motility, and morphology were included as continuous variables, and smoking status [ever (including current and former) vs. never] and abstinence time (≤ 2 days, 3–4 days, ≥ 5 days) were included as categorical variables. Differences between rate ratios with cohort affiliation in the model and those without cohort affiliation in the model were ≤ 10%. There were no changes in *p*-values when cohort affiliation was included; it was not a significant variable and was not retained in the final model. Serum organochlorine concentrations were categorized as tertiles and entered into the model. Concentrations were highly correlated at birth, at age 14, and in adulthood (*r* = 0.77 to *r* = 0.93). To avoid redundant variables and inflated standard errors (Holford et al. 2000; Kleinbaum et al. 2013), organochlorine concentrations were not entered simultaneously. Incidence rate ratios (IRRs) and 95% confidence intervals (CIs) were calculated for each model.

For a subset of men, Pearson correlations were examined for exposures at each of the three time points (adult, 14 years of age, and prenatal). Poisson regression models as described above were constructed for this subset for *p,p´-*DDE and Σ_4_PCBs at the three time points. Because of the possible bias arising from standardizing exposure measures by serum lipids for the adult and age-14 values (Schisterman et al. 2005), models were re-run with lipid concentration as a separate covariate. Because rate ratios calculated with lipid concentration as a separate covariate differed by < 10% and the significance of the *p*-values did not change, the lipid-adjusted results are presented here. All models were run separately using Σ_3_PCBs (138, 153, 180). Because the rate ratios differed by < 10% and the *p*-values were unchanged in the models that included Σ_4_PCBs (118, 138, 153, 180) (data not shown), results are presented here for Σ_4_PCBs only. Complete case analysis was used for calculating means, and observations with missing data were automatically dropped from models. This change affected four observations, bringing the number of observations in the adjusted models to 86. A *p*-value ≤ 0.05 was considered statistically significant.

## Results

Table 1 describes demographic characteristics and semen parameters for the entire sample stratified by cohort affiliation (i.e., birth cohort, fathers cohort, and population register). The men had an average age of 25 years (range: 22–44.5) and a mean BMI of 25.3 kg/m^2^ (range: 19.7–46.1). Slightly more than half of the men (53%) had never smoked. Twelve percent (*n* = 11) had sperm concentrations < 15 million/mL, 2% (*n* = 2) had < 40% progressively motile sperm, and 75% (*n* = 65) had < 4% normally shaped sperm. Of the total 90 men evaluated, 12 were men with proven fertility (fathers cohort). Men with proven fertility were older (mean age 35 years) than other men in the study (mean age 23 years); additionally, more men in the fathers cohort had < 4% normally shaped sperm, and more were smokers.

**Table 1:** Characteristics of adult Faroese men for the total sample and by cohort [*n* (%) or mean ± SD].

Characteristic	Total sample (*n *= 90)	Birth cohort (*n *= 40)	Fathers cohort (*n *= 12)	Population register (*n *= 38)
Age (years)	25.0 ± 4.8	22.6 ± 0.49	35.0 ± 5.6	24.1 ± 0.39
BMI (kg/m^2^)	25.3 ± 4.4	24.8 ± 4.1	26.0 ± 3.8	25.6 ± 4.6
Semen concentration
< 15 million/mL	11 (12. 2)	5 (12.5)	1 (8.3)	5 (13.2)
Motility
< 40% motile	2 (2.2)	1 (2.5)	0 (0)	1 (2.6)
Morphology (*n *= 87)^*a*^
< 4% normal	65 (74.7)	27 (71.1)	10 (83.3)	28 (75.7)
Abstinence time
≤ 2 days	31 (34.4)	14 (35.0)	2 (16.7)	15 (39.5)
3–4 days	44 (48.9)	16 (40.0)	8 (66.7)	20 (52.6)
≥ 5 days	15 (16.7)	10 (25.0)	2 (16.7)	3 (7.9)
Smoking status (*n *= 89)^*b*^
Yes (current/former)	42 (47.2)	17 (42.5)	7 (58.3)	18 (48.6)
No	47 (52.8)	23 (57.5)	5 (41.7)	19 (51.4)
BMI, body mass index. ^***a***^Morphology data were missing for 2 birth cohort participants and 1 population register participant. ^***b***^Smoking data were missing for 1 population register participant.				

The sperm disomy results summarized in Table 2 show that a median of 7,164 sperm nuclei were scored per subject. The observed median percentages of XX18, YY18, XY18, and total disomy were 0.2, 0.26, 0.54, and 1.17, respectively.

**Table 2:** Number of nuclei scored and percent disomy for Faroese men (*n *= 90).

Variable	Mean ± SD	Median	25th percentile	75th percentile
Nuclei (*n*)	7,164 ± 4,011	7,098	4,442	10,093
Percent X18	41.51 ± 6.40	43.09	38.90	46.08
Percent Y18	39.50 ± 5.28	40.74	36.80	43.45
Percent XX18	0.30 ± 0.26	0.20	0.12	0.37
Percent YY18	0.29 ± 0.17	0.26	0.15	0.43
Percent XY18	0.81 ± 0.54	0.54	0.43	1.03
Percent total disomy	1.39 ± 0.79	1.17	0.85	1.69

Table 3 summarizes the distribution of lipid-adjusted *p,p´*-DDE and selected PCB concentrations. The medians for *p,p´-*DDE and Σ_4_PCBs were 0.28 μg/g and 0.39 μg/g, respectively. The serum organochlorine levels were higher in the men of proven fertility (median *p,p´*-DDE of 0.79) than in the other men (median *p,p´*-DDE of 0.25), and the median Σ_4_PCBs was 0.94 in the men with proven fertility, whereas this value was 0.36 in the other men. This finding is consistent with the older age of the fathers cohort, but their semen parameters and disomy rates did not differ significantly from those of the other men (data not shown). Table 3 also summarizes the distribution of *p,p´*-DDE and Σ_4_PCB exposures for the subgroups of men who had prenatal and age-14 exposure measurements. Median values were lowest in the adult sample.

**Table 3:** Distribution of *p,p´*-DDE and PCB exposures for adult Faroese men (*n *= 90).

Exposure	< LOD *n* (%)	Geometric mean	Mean ± SD	Median	Interquartile range (Q25–Q75)
Adult (*n *= 90)
*p,p´*-DDE	0 (0)	0.28	0.51 ± 0.64	0.28	0.13–0.69
Σ_4_PCBs	0 (0)	0.37	0.53 ± 0.48	0.39	0.19–0.65
PCB 118	10 (11.1)	0.02	0.04 ± 0.04	0.02	0.01–0.04
PCB 138	11 (12.2)	0.04	0.12 ± 0.18	0.04	0.01–0.13
PCB 153	0 (0)	0.16	0.22 ± 0.19	0.17	0.09–0.31
PCB 180	0 (0)	0.10	0.15 ± 0.13	0.12	0.05–0.21
Age 14 (*n *= 33)
*p,p´*-DDE	0 (0)	0.79	1.14 ± 1.15	0.72	0.47–1.17
Σ_4_PCBs	0 (0)	0.51	0.66 ± 0.52	0.54	0.32–0.84
Cord blood (*n *= 40)
*p,p’*-DDE	0 (0)	0.45	0.60 ± 0.49	0.43	0.27–0.86
Σ_4_PCBs	0 (0)	0.48	0.59 ± 0.42	0.46	0.35–0.69
Abbreviations: Σ_4_PCBs = sum of PCB 118, 138, 153, and 180; *p*,*p´*-DDE, dichlorodiphenyldichloroethylene; LOD, limit of detection; PCB, polychlorinated biphenyl. Adult and age 14 concentrations μg/g lipid; cord blood concentrations μg/mL. < LOD values for PCB 118 and 138 were imputed before measures of central tendency were estimated.					

Correlations between the prenatal and adult and the prenatal and age-14 log-transformed *p,p´*-DDE values (Table 4) were weak (Pearson’s *r* = 0.20 and 0.22, respectively), whereas the correlation between the age-14 and adult values was moderate (Pearson’s *r* = 0.52). Correlations between the prenatal and adult and the prenatal and age-14 log-transformed Σ_4_PCBs values were weak (Pearson’s *r* = 0.25 and 0.15, respectively), whereas the correlation between the age-14 and adult values was again moderate (Pearson’s *r* = 0.75).

**Table 4:** Pearson correlations (*p*-values) for natural log–transformed organochlorine exposures in cord blood, age 14, and adult samples.

	Age 14 Σ_4_PCB	Adult Σ_4_PCB	Cord blood *p,p’*-DDE	Age 14 *p,p’*-DDE	Adult *p,p’*-DDE
Cord blood Σ_4_PCB	0.15 (0.41)	0.25 (0.12)	0.88 (< 0.0001)	0.10 (0.58)	0.14 (0.40)
Age 14 Σ_4_PCB		0.75 (< 0.001)	0.09 (0.62)	0.77 (< 0.001)	0.59 (0.003)
Adult Σ_4_PCB			0.26 (0.10)	0.54 (0.001)	0.93 (< 0.0001)
Cord blood *p,p’*-DDE				0.22 (0.23)	0.20 (0.21)
Age 14 *p,p’*-DDE					0.52 (0.002)
Adult *p,p’*-DDE
Abbreviations: Σ_4_PCBs = sum of PCB 118, 138, 153, and 180; *p*,*p´*-DDE, dichlorodiphenyldichloroethylene; PCB, polychlorinated biphenyl. Adult and age-14 concentrations, μg/g lipid; cord blood concentrations, μg/mL.					

Tertile cut points were different among the three age groups for *p,p´*-DDE, whereas tertile cut points among the three age groups for PCBs were similar (Table 5). Poisson regression models using exposure tertiles showed that for the adults, *p,p´*-DDE was associated with increased rates of XX18 (IRR = 1.52; 95% CI: 1.35, 1.72), XY18 (IRR = 1.40; 95% CI: 1.30, 1.51), and total disomy (IRR = 1.32; 95% CI: 1.25, 1.35), comparing the highest with the lowest tertile. For YY and Σ_4_PCBs, the IRR increased for tertile 2 (IRR = 1.16; 95% CI: 1.03, 1.32) and decreased for tertile 3 (IRR = 0.85; 95% CI: 0.74, 0.96). Unadjusted results (not shown) were similar to the adjusted results detailed in Table 5. Age-14 exposures showed similar associations between *p,p´*-DDE and sperm disomy to those seen in adults. Age-14 exposure associations between PCBs and sperm disomy were consistently far from the null. Disomy associations with *p,p´*-DDE in cord blood in tertile 2 were consistently negative, in contrast with corresponding associations in adults, which were more likely to be null. Disomy associations with *p,p´*-DDE in cord blood in tertile 3 were generally null, in contrast with corresponding associations in adults, which were generally positive.

**Table 5:** Adjusted*^a^* incidence rate ratios (95% CI) for XX, YY, XY, and total sex-chromosome disomy by *p,p’*-DDE and Σ_4_PCBs tertiles at different time points.

Exposure	Tertile 2	Tertile 3
Cord blood *p,p’*-DDE (*n*= 40)	0.34–0.54 μg/mL	> 0.54 μg/mL
XX18	0.58 (0.48, 0.71)	1.07 (0.87, 1.32)
YY18	0.80 (0.68, 0.94)	0.84 (0.69, 1.02)
XY18	0.67 (0.60, 0.75)	0.97 (0.86, 1.09)
Total disomy	0.68 (0.63, 0.74)	0.94 (0.86, 1.03)
Age 14 *p,p’*-DDE (*n *= 33)	0.54–1.08 μg/g lipid	> 1.08 μg/g lipid
XX18	1.02 (0.77, 1.35)	1.77 (1.39, 2.26)
YY18	0.57 (0.46, 0.71)	0.39 (0.30, 0.50)
XY18	0.85 (0.73, 0.99)	1.45 (1.26, 1.65)
Total disomy	0.79 (0.71, 0.89)	1.19 (1.08, 1.32)
Adult *p,p’*-DDE (*n *= 86)	0.18–0.39 μg/g lipid	> 0.39 μg/g lipid
XX18	0.90 (0.79, 1.03)	1.52 (1.35, 1.72)
YY18	0.88 (0.78, 0.99)	0.93 (0.82, 1.05)
XY18	1.03 (0.95, 1.12)	1.40 (1.30, 1.51)
Total disomy	0.98 (0.92, 1.04)	1.32 (1.25, 1.35)
Cord blood Σ_4_PCBs (*n *= 40)	0.40–0.59 μg/mL	> 0.59 μg/mL
XX18	1.06 (0.86, 1.31)	1.07 (0.87, 1.31)
YY18	1.22 (1.03, 1.45)	0.94 (0.78, 1.14)
XY18	1.08 (0.96, 1.22)	1.16 (1.03, 1.30)
Total disomy	1.09 (0.99, 1.18)	1.09 (0.99, 1.19)
Age 14 Σ_4_PCBs (*n *= 33)	0.40–0.59 μg/g lipid	> 0.59 μg/g lipid
XX18	1.21 (0.82, 1.80)	1.93 (1.42, 2.62)
YY18	1.58 (1.12, 2.21)	0.63 (0.46, 0.86)
XY18	1.66 (1.32, 2.08)	2.23 (1.88, 2.65)
Total disomy	1.64 (1.37, 1.92)	1.81 (1.58, 2.06)
Adult Σ_4_PCBs (*n *= 86)	0.28–0.56 μg/g lipid	> 0.56 μg/g lipid
XX18	0.76 (0.66, 0.87)	1.22 (1.09, 1.38)
YY18	1.16 (1.03, 1.32)	0.85 (0.74, 0.96)
XY18	0.90 (0.83, 0.98)	1.13 (1.05, 1.22)
Total disomy	0.94 (0.88, 1.00)	1.10 (1.04, 1.16)
Abbreviations: Σ_4_PCBs = sum of PCB 118, 138, 153, and 180; *p*,*p´*-DDE, dichlorodiphenyldichloroethylene; PCB, polychlorinated biphenyl. ^***a***^Adjusted for age, abstinence time, smoking status, log(sperm concentration), morphology, and motility. Adult and age-14 concentrations, μg/g lipid; cord blood concentrations, μg/mL.		

Because age differences between the fathers cohort and the other men could have introduced systematic differences, the 12 men from the fathers cohort were removed, and the analyses represented in Table 5 were repeated. The results obtained when these men were omitted showed patterns of statistical significance consistent with those obtained when the men were included (data not shown).

## Discussion

Within this sample of men from the Faroe Islands, there were increases in the rates of an extra X chromosome and total sex-chromosome disomy associated with elevated exposures to *p,p´*-DDE and PCBs that were measured in adulthood, and these results persisted after adjustment for potential confounders. There was evidence of a negative association between *p,p´*-DDE and YY18, whereas results for PCBs and YY18 were inconsistent, with a significant positive association in the second tertile and a significant negative association in the third tertile.

Aneuploidy occurs during the disruption of meiosis during gametogenesis. It is not known how hormone-disrupting pollutants such as *p,p´*-DDE and PCBs interfere with the meiotic phase, but infertile men often have an impaired chromosome synapsis and an increased frequency of chromosomes that are missing a recombination site (Martin 2006; Sun et al. 2008). These errors make the cells susceptible to meiotic arrest and production of aneuploid gametes. Altered recombination affects nondisjunction; non-recombinant chromosomes are susceptible to nondisjunction because of reduced connections among homologous chromosome pairs (Ferguson et al. 2007). Chemicals known to disrupt hormone signaling have been shown to affect mammalian recombination and germ cell aneuploidy (reviewed by Pacchierotti and Eichenlaub-Ritter 2011). Changes in the endocrinologic environment of the testis affect the rate of meiotic segregation errors in mice (Oppedisano et al. 2002), and *p,p*´-DDE has been shown to have an effect on calcium ion channels (CatSper channels), influencing Ca^2+^ increases that in turn affect sperm capacitation, chemotaxis, hyperactivation, and acrosomal exocytosis (Schiffer et al. 2014). Links between organochlorine exposures and early fetal loss (reviewed by Eskenazi et al. 2009) add additional human evidence to the potential links between organochlorines and gametogenesis and reinforce that connections between organochlorine action and chromosome disomy need further *in vivo* and epidemiologic study.

The Faroese birth cohort allowed for a subgroup evaluation of organochlorine exposures at three developmental time points, and positive associations were observed in particular between *p,p´*-DDE and PCB exposures measured at age 14 and adult sperm disomy rates. Because prenatal exposure was determined only from the concentrations in whole blood from the umbilical cord, imprecision in this exposure measurement and the small number of observations may have affected associations of cord blood levels with subsequent sperm disomy measured in adulthood. There is evidence that prenatal exposure to endocrine-disrupting chemicals can predispose adult men to impaired semen quality and testis cancer (McLachlan et al. 1998) and to infertility (Juul et al. 2014), suggesting that early development of testicular cells is particularly vulnerable to adverse exposures. To our knowledge, this is the first study evaluating associations between prenatal and early developmental exposure to these compounds and subsequent sperm chromosomal abnormalities.

In a previous cross-sectional study, we investigated the association of *p,p*´-DDE and the same PCB congener concentrations in serum with sperm sex-chromosome disomy in 192 men from subfertile couples at the Massachusetts General Hospital in Boston, Massachusetts (McAuliffe et al. 2012a). A significant positive trend was found for XX, XY, and total sex-chromosome disomy by increasing quartiles of serum *p,p´-*DDE, and there was evidence of a negative association with YY that was not significant. These positive associations for *p,p´*-DDE with XX, XY, and total disomy and evidence of a negative association with YY disomy were similar to the patterns observed here among men recruited from the community rather than from fertility clinic settings. As in our prior study, our findings for PCBs and XX and YY disomy were less consistent. In our prior study, we saw a significant negative association for PCBs and XX, whereas a positive association was observed in the present study. In our prior study, there was a positive association for YY, whereas in the present study, a positive association was observed in the second exposure tertile and a negative association in the third exposure tertile. These findings may in part be attributable to the accuracy of the frequency estimates because XX and YY have lower frequencies than XY and total disomy.

It is not possible to compare the present results with those of other studies of sperm disomy beyond the previous cross-sectional study, and studies are needed to prospectively evaluate maternal and paternal organochlorine expsoures and risk for Turner or Klinefelter syndromes. The ratio of Y- to X-chromosome–bearing sperm in relation to organochlorine exposures has been measured in sperm and observed from live births (secondary sex ratio). High PCB exposures have been associated with a higher Y:X ratio in sperm among men from Sweden and Greenland (Tiido et al. 2006). Associations between organochlorine exposures and altered secondary sex ratios among live births have been observed to be biphasic, with an increased ratio associated with acute exposure and a decreased ratio associated with persistent and chronic exposures (Hertz-Picciotto et al. 2008; Karmaus et al. 2002).

The Faroe Islands population is exposed to higher levels of persistent organic pollutants than other populations (Longnecker et al. 2003; Weihe and Joensen 2012), and the serum concentrations of *p,p´*-DDE and PCBs (138,153, and 180) measured in this study were much higher than those reported for the U.S. general population in 2003–2004 in the most recent (2009) NHANES report (CDC 2009) and those reported in our previous study (McAuliffe et al. 2012a). For example, the median for *p,p´-*DDE was 200 ng/g lipid in men in the 2003–2004 NHANES, compared with 510 ng/g lipid in the Faroese men. The median for PCB 180 was 18.5 ng/g lipid in the 2003–2004 NHANES men and 150 ng/g lipid in the Faroese men.

The cord blood results were not lipid adjusted, and therefore, exposure misclassification cannot be ruled out. The small sample size did not limit the detection of significant cross-sectional associations in adulthood and separate prospective associations involving exposures measured at age 14. PCBs were highly correlated with *p,p´-*DDE at age 14 and in adulthood, and it was not possible to separate out which time point—age 14 or adulthood—contributed most strongly to this association. Tertile cut points for *p,p´-*DDE were different among the three age groups, limiting dose–response comparisons across time points. There is a potential for bias in the results because of uncontrolled confounding.

## Conclusion

Whereas prior studies investigating environmental exposures and sperm disomy have focused on men seeking evaluation of, or treatment for, infertility, in this study, men were recruited from the general Faroese population. The population’s wide range of organochlorine exposures and homogeneity in demographic and lifestyle characteristics is an advantage for conserving power and reducing confounding. Taken together, the results reported herein further demonstrate links between organochlorine exposures and sperm abnormalities and illustrate that the impacts of persistent pollutants on testicular maturation and function need deeper investigation.
